# Azole Resistance of Environmental and Clinical Aspergillus fumigatus Isolates from Switzerland

**DOI:** 10.1128/AAC.02088-17

**Published:** 2018-03-27

**Authors:** Arnaud Riat, Jérôme Plojoux, Katia Gindro, Jacques Schrenzel, Dominique Sanglard

**Affiliations:** a Service of Infectious Diseases and Service of Laboratory Medicine, Geneva University Hospitals and Geneva University, Geneva, Switzerland; b Division of Pneumology, Geneva University Hospitals, Geneva, Switzerland; c Agroscope, Strategic Research Division Plant Protection, Mycology and Biotechnology, Nyon, Switzerland; d Institute of Microbiology, University of Lausanne and University Hospital Center, Lausanne, Switzerland

**Keywords:** *Aspergillus*, antifungal agents, antifungal resistance, azole

## Abstract

Aspergillus fumigatus is a ubiquitous opportunistic pathogen. This fungus can acquire resistance to azole antifungals due to mutations in the azole target (*cyp51A*). Recently, *cyp51A* mutations typical for environmental azole resistance acquisition (for example, TR_34_/L98H) have been reported. These mutations can also be found in isolates recovered from patients. Environmental azole resistance acquisition has been reported on several continents. Here we describe, for the first time, the occurrence of azole-resistant A. fumigatus isolates of environmental origin in Switzerland with *cyp51A* mutations, and we show that these isolates can also be recovered from a few patients. While the TR_34_/L98H mutation was dominant, a single azole-resistant isolate exhibited a *cyp51A* mutation (G54R) that was reported only for clinical isolates. In conclusion, our study demonstrates that azole resistance with an environmental signature is present in environments and patients of Swiss origin and that mutations believed to be unique to clinical settings are now also observed in the environment.

## INTRODUCTION

Aspergillus fumigatus is a common and widespread filamentous fungus. A. fumigatus can disperse easily in the environment due to its ability to form conidia. Due to their small diameter, conidia can easily reach the lower respiratory airways of humans. This characteristic makes A. fumigatus a cause of several pulmonary diseases in humans, ranging from aspergilloma to allergic bronchopulmonary aspergillosis (ABPA) and various forms of invasive aspergillosis (IA) (1). The treatment of aspergillosis syndrome involves four triazole antifungal drugs: itraconazole (ITZ), voriconazole (VRZ), posaconazole (POS), and isavuconazole (ISA). ITZ was the first orally active drug for aspergillosis (2). VRZ was approved next and showed its superiority to amphotericin B (3). The oral and intravenous availability of VRZ and its positive effects on patients made this drug the first choice for IA in almost all clinical guidelines (4). POS was approved later for the prevention of fungal infection (especially aspergillosis) in leukemia and bone marrow transplant patients and for second-line therapy (5). ISA has been approved more recently for the treatment of IA (6).

The use of antifungal agents is inevitably associated with the occurrence of resistance. After azoles were introduced to treat A. fumigatus infections, reports of resistance started to emerge. The first case of azole-resistant A. fumigatus reported involved a mutation in the *cyp51A* gene, the target of azoles in this organism. This isolate revealed a single mutation in which methionine at the codon position 220 was replaced by arginine (M220R). A similar mutation, M220I, was recovered from a Swedish patient in 1998. Since then, several other single substitutions in *cyp51A* have been identified (7). Single substitutions at codons G54, G138, P216, F219, M220, and G448 have been reported (8). In 2007, Mellado et al. (9) identified a novel type of mutation in several pan-azole-resistant clinical isolates associating a single amino acid substitution in *cyp51A* with a 34-bp tandem repeat in the promoter of the same gene (the TR_34_/L98H mutation). This type of mutation was next found in the environment, raising the possibility that agricultural azole use could facilitate the dissemination of such resistant strains (10). The TR_34_/L98H mutation has now been reported at the scale of several continents (11). In addition, the presence of another mutation, TR_46_/Y121F/T289A, has also been reported in six of nine European countries (12).

Epidemiological data gathered to date show that the frequency of environmental resistance acquisition ranges from 0.5 to 5% in sampled isolates (10). The prevalence of azole resistance in the clinic differs depending on population type and origin, ranging from 4 to 16% (11, 13).

Reports of A. fumigatus azole resistance in Switzerland are rare. A prospective study launched between 2001 and 2003 addressed the occurrence of azole resistance in isolates from clinical and environmental samples. Among the isolates recovered (*n* = 550), only two exhibited ITZ resistance (14, 15). This result was obtained with a neutral selection of isolates from available samples, which were systematically analyzed for azole susceptibility. We report here a novel study in which isolates were recovered from clinical and environmental samples from a small geographical area in Switzerland. Samples were analyzed for the presence of azole-resistant isolates by a widely used positive-selection procedure. Following this, screening for azole-resistant A. fumigatus was implemented at the Geneva University Hospital (HUG), and as a result, two strains of A. fumigatus with TR_34_/L98H were identified in two different patients with cystic fibrosis (CF).

## RESULTS

### Environmental isolates.

Sixty-four samples of different environmental origins comprising a variety of sources (compost sites, public/private gardens, vineyard and agriculture soils, and commercial soil samples) were collected in the western part of Switzerland (the region of Geneva, Lausanne, and Neuchâtel) in a 4-month period (August to December 2015) (see Table S1 in the supplemental material). Six of the 64 samples (9.3%) showed the growth of A. fumigatus colonies on Sabouraud dextrose agar with 4 μg/ml ITZ (SabITZ); 5 A. fumigatus isolates (5d, 15a, 15f, 37c, and 50a) from these samples were VIPcheck positive, confirming that they were ITZ and VRZ resistant. The isolates were further analyzed by matrix-assisted laser desorption ionization—time of flight (MALDI-TOF) mass spectrometry (MS) to confirm their species assignment, and all were identified as A. fumigatus. Susceptibility testing of the A. fumigatus isolates was next performed (by the Sensititre YeastOne [SYO] and CLSI methods) and showed distinct patterns of resistance (Table 1). While all the isolates but one (isolate 61) were ITZ resistant (MIC, >16 μg/ml), the VRZ and POS MICs differed. One isolate (5d) stood out, since it was still susceptible to VRZ (Table 1). Isolate 61 exhibited an ITZ MIC that was low (1 μg/ml) but still higher than that of a susceptible control (isolate 37a, with a MIC of 0.25 μg/ml). The *cyp51A* gene and its promoter region from these isolates were sequenced to support the antifungal susceptibility results. The sequences obtained were identical to published A. fumigatus *cyp51A* sequences. These data and separate β-tubulin sequencing (data not shown) thus confirmed that the isolates belonged to A. fumigatus sensu stricto. As summarized in Table 1, the majority of azole-resistant isolates harbored the TR_34_/L98H mutation. The exception was isolate 5d, with a G54R substitution in *cyp51A*. CLSI MIC readings supported the values obtained by the SYO method with two exceptions. Isolate 5d exhibited a VRZ MIC value of >8 μg/ml by the SYO test but 2 μg/ml by the CLSI method. POS MIC values were, in general, higher for some isolates by the CLSI method (1 μg/ml) than by the SYO test (in the range of 0.25 μg/ml), which, however, still categorized these isolates as POS resistant.

**TABLE 1:** Characteristics of A. fumigatus isolates of this study

Isolate	Origin	Sample type	MIC (μg/ml) by:						*cyp51A* mutation	*CSP1* type
			YeastOne Sensititre test			CLSI M38 method				
			ITZ	VRZ	POS	ITZ	VRZ	POS		
5d	Environment	Commercial vineyard soil mix	>16	0.25	1	>16	0.25	2	G54R	t01
15a	Environment	Compost sample	>16	>8	1	16	2	1	TR_34_/L98H	t02
15f	Environment	Compost sample	>16	4	0.25	>16	4	1	TR_34_/L98H	t11
37c	Environment	Compost sample	>16	2	0.25	16	2	1	TR_34_/L98H	t02
50a	Environment	Soil sample	>16	4	0.25	>16	2	1	TR_34_/L98H	t02
61	Environment	Commercial soil sample	1	0.5	0.125	1	0.5	0.25	TR_34_/L98H	t03
37a	Environment	Compost sample	0.25	0.25	0.06	0.25	0.25	0.125	WT^*a*^	t03
20089320	Patient	Bronchoalveolar lavage fluid	>16	2	0.25	>16	4	1	TR_34_/L98H	t02
17993925	Patient	Bronchoalveolar lavage fluid	>16	4	0.25	>16	4	1	TR_34_/L98H	t01

a WT, wild type.

### Clinical isolates.

Given the detection of azole-resistant isolates in the environment, it was decided to prospectively screen A. fumigatus isolates from clinical samples (160 isolates) (see Table S2 in the supplemental material) for azole resistance at the University Hospital of Geneva during a period of about 1 year (2016–2017). We identified two azole-resistant isolates in the sputum samples of CF patients (see case descriptions below). These isolates exhibited ITZ, VRZ, and POS resistance (MICs, >16 μg/ml, 4 μg/ml, and 0.25 μg/ml, respectively). Sequencing analysis of *cyp51A* and β-tubulin revealed that both isolates exhibited the TR_34_/L98H mutation; they were classified as A. fumigatus sensu stricto.

### *CSP1* typing.

The *CSP1* gene encodes a cell surface protein that is involved in the conidial germination process and in adherence to the extracellular matrix (16). Klaassen and colleagues used *CSP1* in order to categorize A. fumigatus strains (17). The typing nomenclature is based on repeat regions found in *CSP1*, which are classified into 18 *CSP1* types. It has been suggested that CSP typing, with a lower discriminatory power than microsatellite analysis, is suitable for typing at the subpopulation level (16). We addressed the relationships among our sampled isolates by this method. *CSP1* typing of all isolates showed four different *CSP1* types: the TR_34_/L98H isolates were distributed over the t01, t02, t03, and t11 subtypes, while the G54R isolate belonged to the t01 subtype (Table 1). The azole-susceptible isolate of environmental origin included here belonged to *CSP1* subtype t03.

## DISCUSSION

A. fumigatus harboring the TR_34_/L98H mutation, leading to panazole resistance, was first described in the Netherlands in 2008, followed by other countries (7, 12). The overuse of azoles in agriculture was proposed to be the route of selective pressure on A. fumigatus to develop resistance. In addition, Snelders et al. (18) showed by microsatellite typing that *cyp51A* mutant strains recovered from the environment and from clinical samples clustered together. This likely indicates common ancestors for the two groups of isolates.

The results presented here confirmed previous findings on the occurrence of azole resistance in A. fumigatus (19). Our study confirms the presence of isolates with the TR_34_/L98H mutation in the environment in the western part of Switzerland. Interestingly, a single substitution mutation (G54R) was detected in an environmental A. fumigatus isolate. Mutations at position G54 are known to be the consequence of long-term azole therapy in patients (19). Two recent publications have also shown the presence of single substitutions in *cyp51A* from environmental isolates. The G54E mutation was reported for isolates from environmental samples in Romania, India, and Tanzania, and a novel substitution, G54A, was reported in Germany (20, 21). Since the selective agar medium SabITZ contains 4 μg/ml ITZ, the selection of resistant isolates for which MICs are higher than this concentration can be expected. The TR_34_/L98H and G54R mutations result effectively in high ITZ MICs (>16 μg/ml). The type of *cyp51A* mutation has a differential impact on VRZ MICs: while the TR_34_/L98H mutation invariably results in VRZ resistance (all isolates exhibit MIC values of >2 μg/ml), the G54R mutation does not result in VRZ resistance (MIC, 0.25 μg/ml). Interestingly, Bader and colleagues showed that a selective agar medium with a lower ITZ concentration (1 μg/ml) could identify a broader spectrum of mutations (i.e., TR_46_/Y121F/T289A and TR_46_/Y121F/M172I/T289A), which are associated with a lower ITZ MIC (<2 μg/ml) but with the same VRZ and POS MICs (21). Even though the most prevalent environmental mutation is TR_34_/L98H, the detection of other *cyp51A* mutations is important. Therefore, the choice of an appropriate selective medium will have a direct impact on the screening of resistance in A. fumigatus.

Susceptibility testing was performed by two methods, which agreed with each other for most recorded values. POS MICs for TR_34_/L98H isolates were, in general, higher by the CLSI method than by the SYO test. This difference could be due to endpoint reading issues in the SYO approach, which is based on a change in the color of the metabolic indicator (alamarBlue) rather than on visual inspection of turbidity as performed in the CLSI method. Our antifungal susceptibility testing results were comparable to those of previous studies except for one isolate. This particular isolate (isolate 61), with an ITZ MIC of 1 μg/ml by both methods, was interpreted as susceptible according to the CLSI breakpoints. These results reveal that the TR_34_/L98H mutation is not always correlated with panazole resistance, a pattern consistent with that of a previous study in which the occurrence of TR_34_/L98H was not strictly associated with ITZ resistance (MIC cutoff, 1 μg/ml) (22). Therefore, the azole susceptibility profile could not be inferred from the detection of the TR_34_/L98H mutation alone.

In order to assess the relationships among azole-resistant A. fumigatus isolates, two genotyping methods have generally been applied: microsatellite (STRAf) genotyping and *CSP1* typing. We identified *CSP1* types (t02 and t011) that had already been reported for azole-resistant isolates (21). A. fumigatus isolate 5d (G54R) had *CSP1* type t01, which was also associated with a G54W mutation in the study of Bader et al. (21). However, there seems to be no strict association of the *CSP1* type with the occurrence of resistance, since CSP type t01 is also found in wild-type isolates (17). In any case, the small number of azole-resistant A. fumigatus isolates in our study cannot support the conclusion that these isolates have a common origin.

The presence of azole-resistant A. fumigatus isolates from patients has been monitored in different parts of the world with different levels of prevalence (23). In 2015, Switzerland participated in the SCARE study (23) using isolates originating from a single hospital center (University of Lausanne). Among the 236 isolates tested, none exhibited azole resistance (23). An older study performed in Switzerland using A. fumigatus isolates mostly from CF patients (*n* = 400) but originating from different institutions (all before the year 2003) reported a single ITZ-resistant isolate, which was devoid of the TR_34_/L98H mutation (15). The present study reveals, for the first time in Switzerland, pan-azole-resistant A. fumigatus isolates from CF patients. One particularly noteworthy point was that the records of these patients did not report the use of azoles. This suggests that the source of the azole-resistant isolates may be environmental. This hypothesis was already suggested in a study investigating A. fumigatus azole resistance in CF patients, some of whom were colonized with azole-resistant isolates without prior azole treatment (24).

The isolation of azole-resistant isolates in the Swiss environment is not a surprise *per se*, given that most European countries have reported the same phenomenon. The worldwide occurrence of azole resistance in A. fumigatus poses several issues, one of which is the management of patients with prior colonization by resistant isolates. Such patients should be identified early enough for the rapid adaptation of antifungal therapy. Continuing surveillance of azole resistance should also be more systematically organized in Switzerland than it is currently (absence of surveillance program), and the medical sector should be kept well informed on the progression of resistance at a nation-wide scale.

## MATERIALS AND METHODS

### Environmental samples.

Sixty-four samples collected from different environments in the Lemanic region (Table S1 in the supplemental material) were positively screened for A. fumigatus ITZ resistance using a Sabouraud dextrose agar plate (2%; Becton, Dickinson [BD] Difco) supplemented with ITZ at 4 μg/ml (SabITZ) (Sigma-Aldrich). Each sample was treated as follows before the azole resistance screening procedure. A 1- to 2-g portion of each sample was suspended in an 8-ml solution containing sterile water, 1% Tween 20 (Sigma-Aldrich), and 0.5 g/liter chloramphenicol (Sigma-Aldrich) (25). Each sample was vortexed for 1 min and was then sedimented for 3 h at room temperature according to published recommendations (25). One hundred microliters of the supernatant from the sedimented solution was plated on two Sabouraud dextrose agar plates (2%; BD Difco) and two SabITZ plates (Sigma-Aldrich). The plates were incubated at 37°C for 72 h and were checked for the growth of Aspergillus complex spp. Fungal colonies isolated on SabITZ and Sabouraud agar plates were analyzed with a Microflex LT MALDI-TOF MS (Bruker Daltonics) and were identified as A. fumigatus complex with Biotyper software, version 3.0, using the Filamentous Fungi Library, version 1.0 (Bruker Daltonics). Colonies growing on SabITZ were next screened for resistance using the VIPcheck test, which consists of a 4-well plate containing agar supplemented with three azole drugs (VRZ at 2 μg/ml, ITZ at 4 μg/ml, and POS at 0.5 μg/ml) and a growth control. The plates were incubated at 37°C for 72 h. A. fumigatus isolates with growth on SabITZ and the VIPcheck test were tested for their susceptibility profiles.

### Clinical samples.

A. fumigatus group species isolated from respiratory samples of CF patients collected mostly during the year 2016–2017 (Table S2 in the supplemental material) were tested for azole resistance. Each isolate was recovered from −80°C stocks and was grown on Sabouraud maltose agar. The azole resistance pattern was phenotypically tested using the VIPcheck method. Identification of filamentous fungi was carried out on the Microflex LT MALDI-TOF MS (Bruker Daltonics).

### Susceptibility testing.

A. fumigatus isolates harboring phenotypic resistance to an azole(s) were tested for their susceptibility profiles by the standardized colorimetric microdilution test method, Sensititre YeastOne (SYO) (Thermo Scientific). The test yields MICs for 10 antifungal agents, including ITZ, VRZ, POS, fluconazole, caspofungin, micafungin, anidulafungin, flucytosine, and amphotericin B. The test was performed according to the manufacturer's instructions. Briefly, 100 μl from a 0.5 McFarland standard inoculum of A. fumigatus was added to the SYO broth, which contains RPMI medium 1640 (Thermo Scientific), and was vortexed; then 100 μl of the SYO broth was added to each of the 96 wells of the SYO plate. The SYO plate was incubated at 37°C for 48 to 72 h. MICs were determined by changes in the color of alamarBlue as an indicator of metabolic activity.

The susceptibility of A. fumigatus isolates was also determined according to the CLSI M38-A2 method (26). The MIC was defined as the antifungal concentration at which visual growth was completely inhibited. The isolate was regarded as susceptible when the MIC was <2 μg/ml for ITZ and VRC and <0.25 μg/ml for POS, according to the recommendations of Espinel-Ingroff et al. (27). An isolate containing the known *cyp51A* TR_34_/L98H mutation (CM2627) was included as a control in susceptibility assays (9).

### Sequencing.

Isolates with a phenotypic resistance pattern were sequenced for the *cyp51A* gene. A. fumigatus DNA was extracted using a one-step procedure. The fungal biomass was added to a microcentrifuge tube containing 0.3 g of glass beads (diameter, 0.5 mm), 200 μl phenol-chloroform (Sigma), and 200 μl of breaking buffer (2% Triton X-100, 1% sodium dodecyl sulfate, 10 mM Tris-HCl [pH 8.0], 1 mM EDTA, 100 mM NaCl). After 15 s of shaking at 4,500 rpm using a Precellys Evolution tissue homogenizer (Bertin Instruments, France), samples were centrifuged for 10 min at 13,000 rpm. One microliter of the supernatant was used for PCR.

The *cyp51A* gene was amplified by PCR using two *cyp51A* primers (5′-GAGCCGAATGAAAGTTGCCTAATTACTA-3′ and 5′-CCACAGTTTAGATAGGCTAGAAGGAG-3′). PCR was performed in a 50-μl volume containing 5 μl buffer (ThermoPol reaction buffer; New England BioLabs), 1 μl deoxynucleoside triphosphates (dNTPs) at 10 mM, 1 μl of each primer at 100 μM, 0.5 μl *Taq* polymerase at 5,000 U/ml (New England BioLabs), and 1 μl of lysed supernatant containing genomic DNA. PCR products were purified using ExoSAP-IT reagent (Affymetrix). Five microliters of the PCR product was mixed with 2 μl of ExoSAP-IT reagent. The solution was incubated for 15 min at 37°C, followed by a 15-min incubation at 80°C.

PCR products were next sequenced using two sets of primers: CYP51A-A7 (5′-TCATATGTTGCTCAGCGG-3′) and CYP51A-A5 (5′-TCTCTGCACGCAAAGAAGAAC-3′) for tandem repeat detection and Cyp51a_sq3 (5′-CATGTGCGCAATCTCTTTATC-3′) and Cyp51a_q4 (5′-CGGAAGATAGGGACTTGACGT-3′) for single-site mutation detection. Sequencing used the BigDye Terminator kit (Applied Biosystems, USA). The sequences were analyzed using Geneious software, version 9.16 (Biomatters, New Zealand).

The A. fumigatus β-tubulin gene was amplified with primers tubulin_s (5′-GCTCTGGCCACTACAATGGCT-3′) and tubulin_as (5′-GTCCATGGTACCAGGCTCGA-3′). Gene products were purified and sequenced as described above.

### *CSP1* typing.

A. fumigatus isolates were genotyped by *CSP1* typing. This subtyping strategy employs comparative DNA sequence analysis of the tandem repeat region of the AFUA_3G08890 gene (17). The *CSP1* gene was amplified by PCR using two *CSP1* primers proposed by Klaassen et al. (17): 5′-TTGGGTGGCATTGTGCCAA-3′ and 5′-GAGCATGACAACCCAGATACCA-3′. After PCR, the products were sequenced as described above using the same primers as those used for the amplification. Geneious software was used to analyze results.

### Case reports.

Case 1 concerns a 52-year-old woman. She was diagnosed with homozygous Phe-508del mutation CF when she was 38 years old. She is known to have severe airflow obstruction (predicted forced expiratory volume in 1 second [FEV1] value, 31%) with severe diffuse bronchiectasis and chronic infection by methicillin-susceptible Staphylococcus aureus (MSSA), Achromobacter xylosoxidans, and Stenotrophomonas maltophilia. This chronic respiratory infection is complicated by acute respiratory exacerbations necessitating intravenous antibiotic cures 2 to 3 times per year. Additionally, she recently received a successful 18-month course of antimycobacterial medication (rifabutin, clarithromycin, and ethambutol) for Mycobacterium avium-associated pulmonary disease. She has demonstrated chronic respiratory colonization with A. fumigatus since CF diagnosis but has never developed active Aspergillus infection or ABPA. Her total IgE level is undetectable. She has never received antifungal therapy, including triazoles. Her regular treatment is based on oral pancreatin, liposoluble vitamin supplementation, and ursodeoxycholic acid and inhaled budesonide, formoterol, and dornase alpha. In addition, she follows a twice-weekly physical treatment program for respiratory secretion drainage with a physiotherapist. Since the patient is already presenting severe alteration in lung function, which is still declining, double lung transplantation will be considered mandatory in the coming years.

Case 2 concerns a 27-year-old man. He was diagnosed with homozygous Phe-508del mutation CF when he was born due to meconium ileus, requiring surgery. He is known to have stable mild airflow obstruction (predicted FEV1 value, 82%) and mild diffuse bronchiectasis with chronic respiratory colonization by MSSA, Stenotrophomonas maltophilia, and Haemophilus influenzae. Acute respiratory exacerbations are treated with an oral course of antibiotics against MSSA 1 to 2 times per year. He has demonstrated new chronic respiratory colonization with A. fumigatus since January 2016. He has never developed active aspergillus infection or ABPA. His total IgE level measurements have remained between 15 and 21.9 kU/liter during the past 24 months (normal value, <100 kU/liter). He has never received antifungal therapy, including triazoles. His treatment is based on oral pancreatin, liposoluble vitamin supplementation, and ursodeoxycholic acid and inhaled fluticasone propionate and salbutamol. In addition, he follows a weekly physical treatment program for respiratory secretion drainage with a physiotherapist.

## Supplementary Material

Supplemental material
