# Supplementary material for: Azole Resistance of Environmental and Clinical Aspergillus fumigatus Isolates from Switzerland
Source: Antimicrob Agents Chemother. 2018 Mar 27;62(4):e02088-17. doi: 10.1128/AAC.02088-17 (PMC5913999; doi:10.1128/AAC.02088-17)
Supplement: Supplemental material [file supp_62_4_e02088-17__index.html]

Azole Resistance of Environmental and Clinical Aspergillus fumigatus Isolates from Switzerland — Supplemental material 

# Azole Resistance of Environmental and Clinical Aspergillus fumigatus Isolates from Switzerland

## Supplemental material

- Supplemental file 1 -

  TABLE S1

  XLSX, 41K
- Supplemental file 2 -

  TABLE S2

  XLSX, 54K
